# Attitudes of Palestinian medical students on the geopolitical barriers to accessing hospitals for clinical training: a qualitative study

**DOI:** 10.1186/s13031-016-0067-8

**Published:** 2016-02-24

**Authors:** Sarrah Shahawy, Megan Diamond

**Affiliations:** Harvard Medical School, 25 Shattuck St, Boston, MA 02115 USA; Harvard T.H. Chan School of Public Health, 677 Huntington Ave, Boston, MA 02115 USA

**Keywords:** Occupation, Palestine, Medical education, West Bank, Al-Quds University, Medical students

## Abstract

**Background:**

The movement of Palestinians in the occupied Palestinian territories is restricted by bureaucratic and physical obstacles. To date, no studies have examined the barriers that Palestinian medical students face in accessing hospitals for clinical training. The objectives of this study were to characterize these barriers and understand how they affect Palestinian students’ medical education and quality of life.

**Methods:**

Convenience sampling was used to recruit 4th-6th year medical students from Al-Quds University to participate in focus group discussions. A total of 36 students participated in the discussions. Transcripts of the discussions were coded to identify major themes.

**Results:**

Palestinian medical students expressed facing numerous challenges during their clinical training. Students emphasized the difficulties of obtaining permits to train at Jerusalem hospitals, including arbitrary permit rejections and long wait times. Significant delays, searches, and mistreatment at checkpoints during their commute to hospitals were particularly burdensome. The majority of students who participated in the focus groups felt that their education and quality of life had been strongly negatively affected by their experience trying to access hospital training sites.

**Conclusions:**

Our findings suggest that medical students living and studying in the occupied Palestinian territories receive sub-optimal training due to ambiguous permit rules, barriers at checkpoints, and the psychological burden of the process. These results highlight the impact that military occupation has on the education and quality of life of Palestinian medical students in a setting in which there is regular violence and many health indicators are already poor.

**Electronic supplementary material:**

The online version of this article (doi:10.1186/s13031-016-0067-8) contains supplementary material, which is available to authorized users.

## Background

The history of the occupied Palestinian territories (OPT), which consist of the West Bank, East Jerusalem and the Gaza Strip, has been turbulent. Due to a military occupation that began in 1967, the movement of Palestinians within the occupied territories is constantly restricted [1, 2]. Palestinians in the OPT can carry one of 3 ID types, each of which defines where they are allowed to freely travel within the region. The Jerusalem ID allows holders to reside in Jerusalem and enter the West Bank; the West Bank ID prevents those living in the West Bank from entering Jerusalem without applying for special permission; and the Gaza ID prevents residents from leaving Gaza. Beyond ID status, movement is largely restricted within and from the West Bank by a complex series of bureaucratic and physical obstacles, including the need for special permits to enter Jerusalem, military checkpoints, roads forbidden to Palestinians, and an eight-meter high, 700 km-long concrete Separation Wall that separates the West Bank from Jerusalem and Israel [3]. In 2015, there were 96 permanent military checkpoints within the West Bank, 361 temporary “surprise” checkpoints monthly, and 39 checkpoints that regulate movement out of the West Bank and into Jerusalem and other cities [4, 5].

The current geopolitical context of the OPT poses challenges to healthcare delivery and access [1, 6]. The OPT has a fragmented landscape of healthcare providers with hospitals representing a mix of governmental, private, and non-governmental organization (NGO) entities [3]. The Palestinian hospitals in East Jerusalem are considered the most advanced in the OPT and for decades, they have served the people of the West Bank and Gaza, especially for complex cases or medical specialties unavailable locally [7]. The tertiary hospital for the OPT, Al-Makassed Hospital, serves over 60 % of the population and is located in East Jerusalem. The construction of the West Bank Separation Wall beginning in 2002 has made the hospital difficult to access by Palestinian patients and health care staff living in the West Bank or Gaza due to the need for permits to enter Jerusalem and because of the arduous commutes through checkpoints [7, 8]. These obstacles contribute to poor health in the OPT [6] and may potentially restrict the education and clinical training of Palestinian medical students.

**Fig. 1:**
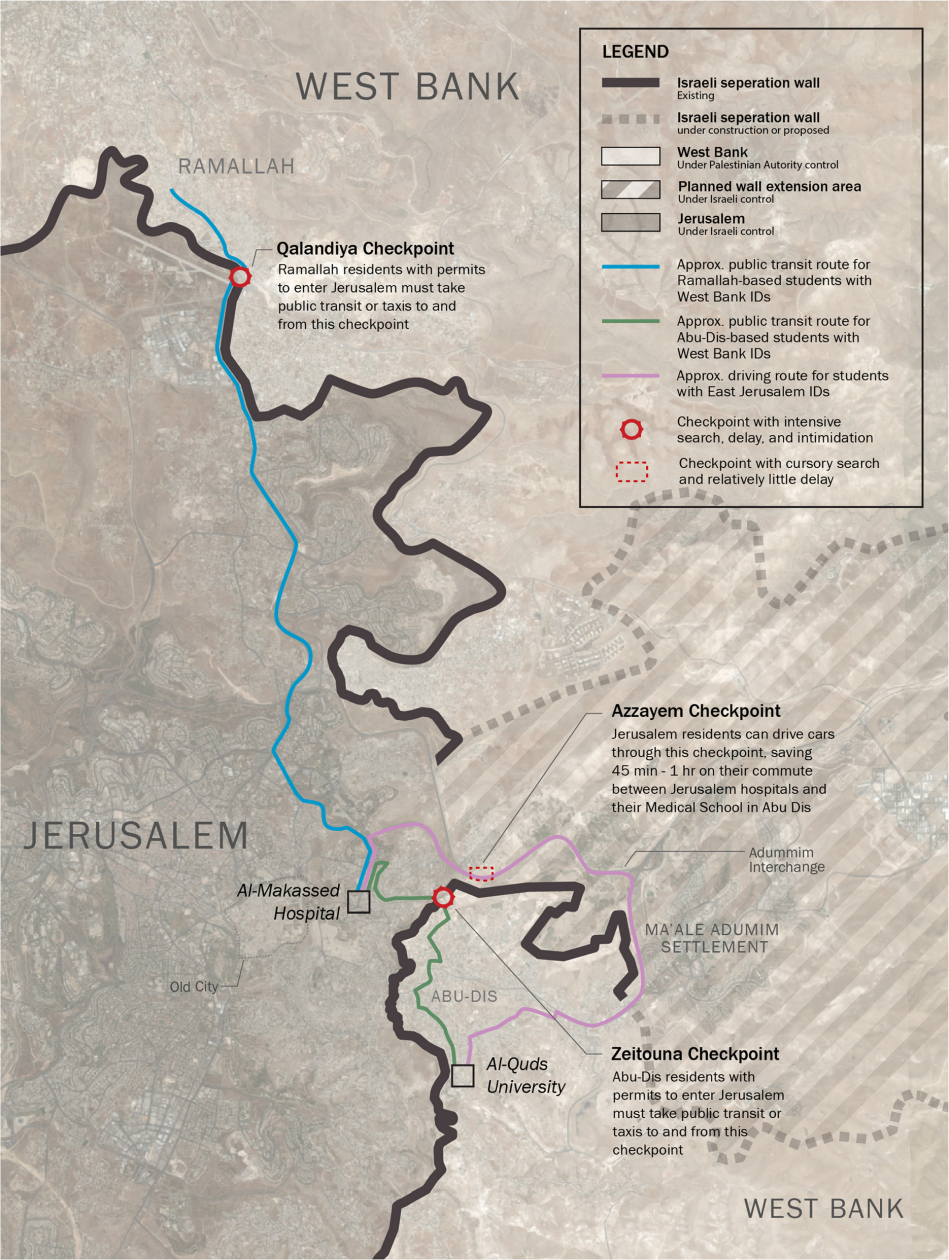
Map of Palestinian medical students’ routes to Jerusalem hospitals. Shown are several routes that Palestinian medical students at Al-Quds University in Abu Dis take to commute to one of their main teaching hospitals, Al-Makkased Hospital, located in East Jerusalem. Most medical students at Al-Quds carry West Bank IDs and must take public transit or taxis to and from checkpoints along the Separation Wall that separates their homes in the West Bank from teaching hospitals in Jerusalem. The green route shows how students who live in dormitories at Al-Quds University in the West Bank travel by bus to Zeitouna checkpoint, through which they walk and are searched by Israeli soldiers. They then take another bus or taxi from the checkpoint to Al-Makassed Hospital. The blue route shows how students living in Ramallah, one of the biggest cities in the West Bank, travel by bus to Qalandiya checkpoint, then take another bus or taxi from Qalandiya to the hospital. In contrast, the minority of medical students living in Jerusalem with a Jerusalem ID can more readily reach the hospital and their University with their own cars along well-paved highways (shown by the purple route). *Map by Aaron Reiss*

There are 4 medical schools in the OPT, the premier of which is Al-Quds University Medical School in Abu Dis, two miles east of Jerusalem. Palestinian medical students at Al-Quds rely heavily on the teaching hospitals of East Jerusalem, namely Al-Makassed Hospital, for their core clinical training. Figure 1 shows a map of the OPT and the routes Palestinians must take through checkpoints at the Separation Wall to enter Jerusalem.

In addition to Al-Makassed Hospital, there are three other hospitals at which Al-Quds University medical students can rotate in Jerusalem. In the West Bank, the main hospitals at which students rotate include Al-Ahli Hospital in Hebron, Red Crescent Hospital in Hebron, and the Palestine Health Complex in Ramallah. There is a shortage of tertiary care centers in the West Bank [9]. Al-Makassed Hospital, the main tertiary hospital of the OPT, has strong affiliations with Al-Quds University and serves as the unofficial teaching hospital for medical students. Many of the medical school professors and directors of the clinical clerkships are based at Al-Makassed. Therefore, the curriculum and teaching at Al-Makassed tends to be the most geared towards training medical students. Unlike most hospitals in the West Bank, Al-Makassed has training programs in sub-specialties and is thus a strongly desired location for clerkship training among medical students.

To date, no qualitative studies have specifically examined the barriers Palestinian medical students face in the clinical stages of their training due to the geopolitical context in which they study and work. Thus, the objectives of this study were to characterize these barriers and better understand how these circumstances affect students' medical education and quality of life.

## Methods

We designed a qualitative study using focus group discussions to explore the attitudes of Palestinian medical students at Al-Quds University on the barriers they face reaching hospitals for clinical training.

### Topic guide

The topic guide for the focus group discussions was initially developed through informal conversations prior to the start of the study with Palestinian medical students and Palestinian physicians familiar with the medical education system. The topic guide was then further developed to explore salient points and reviewed by faculty at Harvard Medical School as well as Palestinian physicians and researchers working at the West Bank and Gaza Office of the World Health Organization. The topic guide can be found in the Additional file 1.

### Sampling

A convenience sample was taken from the 215 medical students in their clinical years (years 4–6) at Al-Quds University. There was an equal proportion of males and females in the medical school. Students were recruited from November to December 2014 to participate in focus group discussions.

Students were made aware of the study by announcements and information sheets provided at the end of lectures, through student body representatives providing members with study information sheets, and an email from the Secretary of Student Affairs.

### Focus group discussions

Out of 215 eligible medical students, a total of 36 (16.7 %) students consented to participate in the focus group discussions. Each focus group discussion was divided into four topic areas: demographic characteristics, identification cards and permits, the commute to hospitals, and the effect of delays on their education and quality of life. The discussions were in a semi-structured format, giving participants an opportunity to guide the discussion. Focus groups ranged between 2 to 6 medical students. All interviews were carried out in Arabic by the first author and voice recorded.

The first author and a consultant translator then independently listened to the voice recordings of each focus group discussion and translated them entirely to English to produce written transcripts. Translated transcripts were then compared and agreements on any points of discrepancy were made to produce one final English transcript for each discussion. Both the first author and consultant are fluent in Arabic and English.

### Qualitative data analysis

The two authors open-coded the transcripts using thematic analysis to identify patterns in the focus group discussions. Codes were identified independently then refined collaboratively to finalize a list of reoccurring themes. All coding was done using MAXQDA 11. Key themes and illustrative quotations were reviewed for consensus by the authors.

### Ethics, consent, and permissions

This study was approved by the Harvard Medical School IRB Ethical Review Board (IRB14-3871) and the local Palestinian IRB at Al-Quds University Medical School. Informed consent was obtained verbally with participants provided with a study information sheet. No compensation or incentives were given to study participants. Names of participants were not recorded. All transcribed files and audio recordings were saved on an encrypted password-protected laptop.

## Results

Characteristics of the study population are presented in Table 1. According to the Al-Quds University administration, there were a total of 193 students in years 4–6 in 2014 with a West Bank ID who applied for permits, 13 (6.7 %) of whom were denied.

**Table 1:** Participant characteristics

Characteristic	Focus group participants (*n*=36)
Age (years)	22.7 (±0.9)
Sex	
Male	17 (47.2 %)
Female	19 (52.8 %)
Year of medical school	
4th year	0 (0.0 %)
5th year	24 (66.7 %)
6th year	12 (33.3 %)
ID card type	
West Bank ID	35 (97.2 %)
Jerusalem ID	1 (3.8 %)
Permit to Jerusalem denied	6 (17.1 %) ^a^

^a^ Out of 35 respondents

Thirty-six medical students participated in 9 focus groups ranging in size from 2 to 6 persons. Slightly more females participated (52.8 %) than males. Sixty-seven percent of focus group participants were in their fifth year of training and the remaining were in their sixth year. The vast majority (97.2 %) had a West Bank ID and of those, 17.1 % (6/35) had been denied access to Jerusalem hospitals. An equal number of males and females had their permits denied. The average age of participants was 22 years old.

**Fig. 2:**
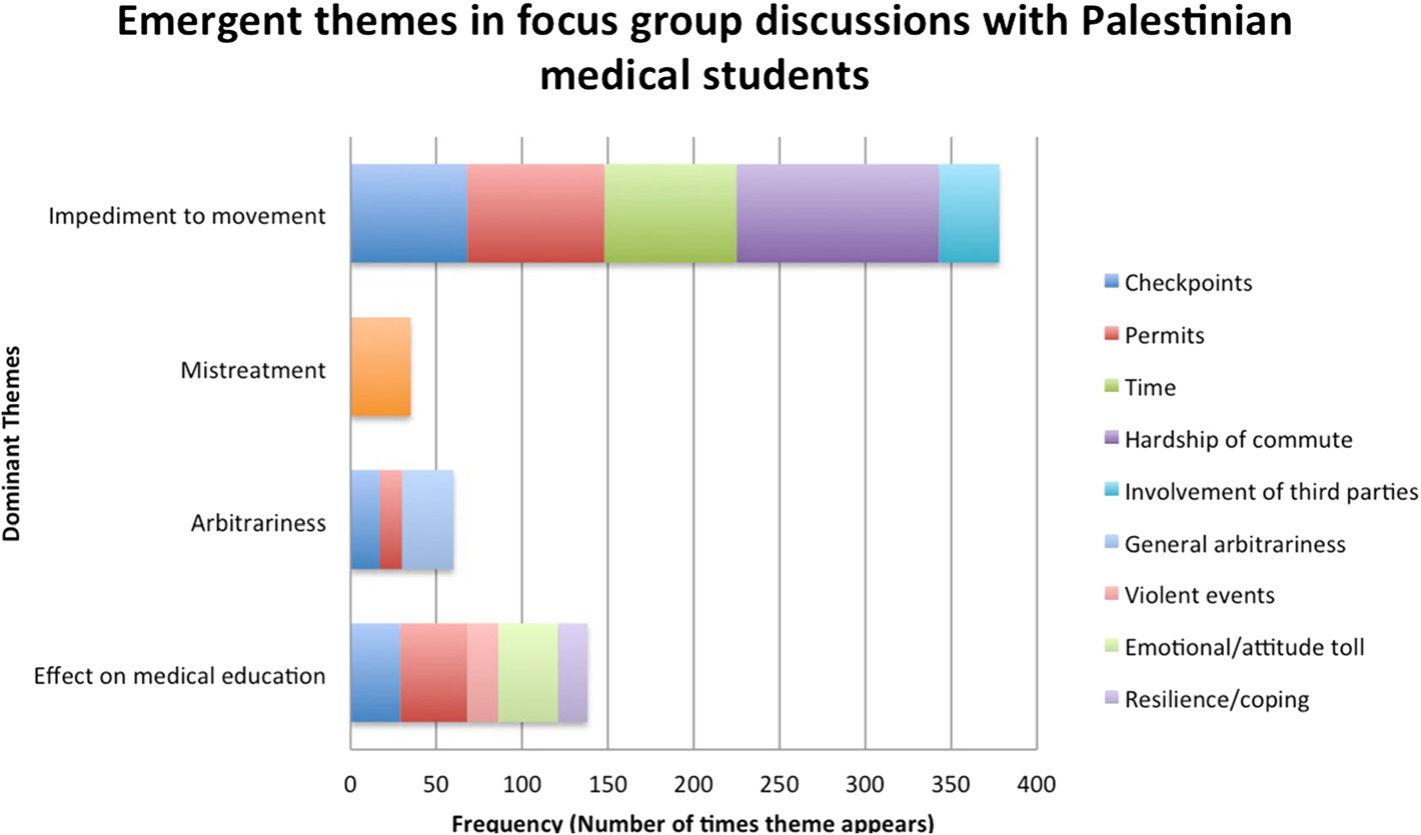
Emergent themes in focus group discussions. The four main themes identified in focus group discussions were further broken down into sub-codes (listed in the legend on the right). The frequency of each sub-code within each of the four dominant themes is shown in the graph

In the focus group discussions, 35 codes were identified that fell under 4 main themes: impediment to movement, mistreatment, arbitrariness, and effect on medical education. An overview of these overarching themes, which were further divided into 13 nuanced sub-codes, is displayed in Fig. 2.

### Impediment to movement

In all focus groups, the issue of impediment to movement within the West Bank and between Jerusalem was the most dominant theme. Movement was primarily restricted by the need for permits, delays and searches at checkpoints, and checkpoint closures.

#### Permits

**Fig. 3:**
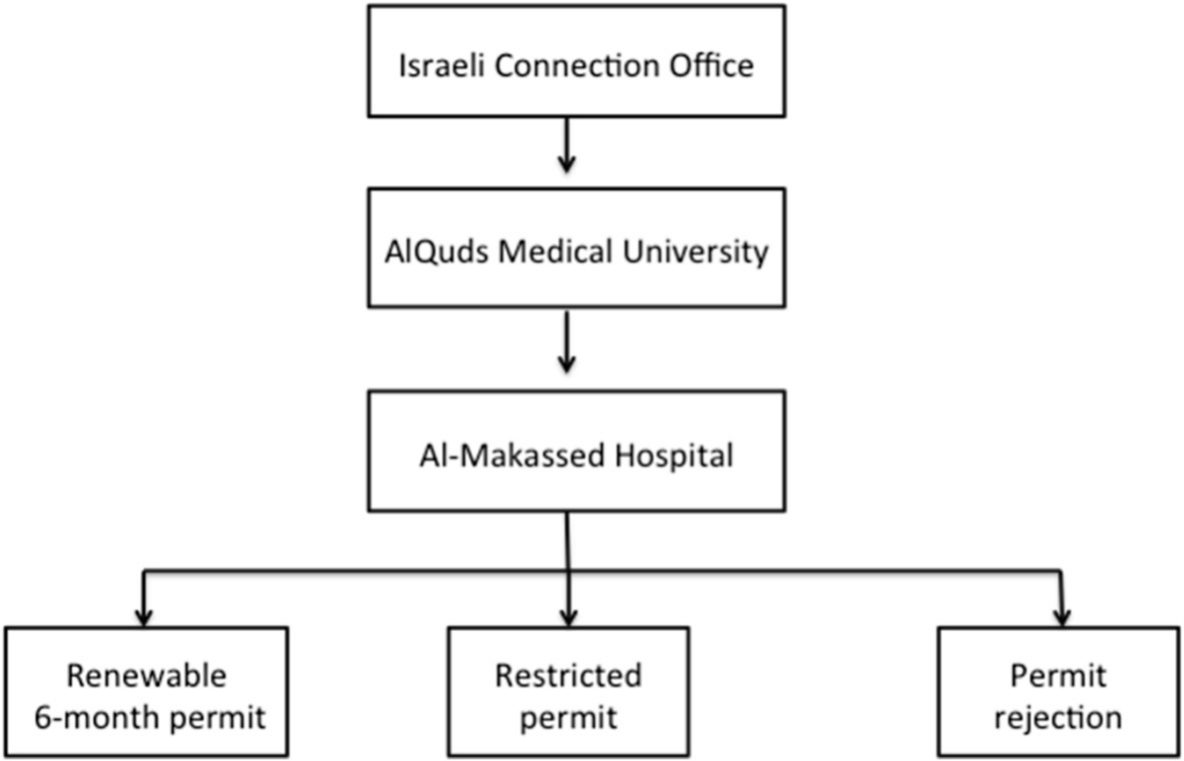
Permit application process for medical students at AlQuds University. Students present their personal ID’s to obtain a Magnetic Card at the Israeli Connection Office. Then, they give their personal IDs and Magnetic Cards to the administration at Al-Quds University who pass it to Al-Makassed Hospital. The hospital administration applies to the Israeli authorities for permits on behalf of students and they are either given a renewable permit, given a restricted permit, or rejected

A description of the process for students with a West Bank ID to obtain a permit to enter Jerusalem is presented in Fig. 3. Students first present their personal ID’s to obtain a Magnetic Card at the Israeli Connection Office in their district. Next, the students give their personal ID and Magnetic Cards to the administration at Al-Quds University who pass it to the Al-Makassed Hospital administration, which applies to the Israeli authorities for permits on behalf of students. Approximately a month later, students are then either given a renewable 6-month permit, which allows students access to the hospitals in Jerusalem at all times and is automatically renewable every 6 months, given a restricted permit, or are rejected. Students whose permits are rejected are unable to enter Jerusalem and are thus unable to rotate at any of the Jerusalem hospitals, including their main unofficial teaching hospital, Al-Makassed.

Students regularly mentioned the amount of time that it took to obtain a permit, which is required before their fourth year of medical school in order to rotate at various teaching hospitals in Jerusalem. Five students in the focus groups noted that they waited over five hours at the Israeli Connection Office in their district before getting a turn to obtain a Magnetic Card. Having to go back and wait multiple times due to the office closures was frequently mentioned. One student, talking about her second attempt at getting a Magnetic Card said, “I waited 6–7 h then another day, it was the same thing from 7 am until 1 pm. It takes a whole day. It takes a lot to get it”. Students also noted that they had to pay to apply for a Magnetic Card, even if their permit applications would later be rejected.

Focus group discussions strongly emphasized the limitations of restricted permits, which only allow students to be in Jerusalem for certain hours of the day and must be renewed more frequently than standard permits. Students also noted that having a restricted permit put them at a higher risk of being searched at checkpoints. One student described the experience of a colleague with a restricted permit: “He gets searched every single day and gets severely mistreated daily, it would have been better if he was not given a permit”. 

#### Hardship of commute

Delays at checkpoints, which included traffic and searches, to and from hospitals in Jerusalem and the West Bank emerged as a common daily impediment for almost all medical students interviewed, regardless of permit or ID status. Discussing the amount of time spent commuting, students reported waiting hours at checkpoints, depending on the number of people in the queue, the practices of the Israeli authorities, and whether there were violent activities occurring nearby. Describing the commute to Al-Makassed Hospital, one student said, “We can waste an hour trying to reach the hospital. The hour itself is not that important, we can waste it on TV…but it causes us stress…2 h all for 2 km…[we are] tired to death not from the rotation but also from the commute”. 

Students were also subjected to searches at checkpoints by Israeli soldiers. As one student describes, “We go through multiple stations, they check and search you. It takes a lot of time, we have to wait…The female soldiers give us a harder time…they are more vicious. They search us. The doors will open and close behind us. There’s a metal detector. We have to take off everything…They make me take off my shoes sometimes”. Another student recalled an experience at a checkpoint: “We once brought a knife for a cake, and they [Israeli soldiers] wouldn’t let us pass and they kept us in a room and interrogated us. A soldier comes in and asks you and investigates you and takes your name. They keep what you took in and let you go, depending on their mood”. Beyond being time consuming, many students mentioned that the daily search process was stressful and one of the main reasons they avoided rotating at hospitals that would require crossing checkpoints.

### Mistreatment

In all focus groups, the issue of mistreatment emerged as a dominant theme. The process of being searched at a checkpoint was felt to be humiliating by many students. As one student describes, “We go through those metal detectors and bars, […] like the ones animals would go through”. Students used expressions such as “humiliated”, “disrespected”, “treated very badly”, and “demeaning” to describe their experience with Israeli soldiers. Another student said, “One female soldier spent half an hour making me wait so that I would say that the place I was going into was ‘Israel’. Tell me, where is Israel? What are its borders? She asked me, ‘Where are you from?’ I said Nablus. She said, ‘There is no Nablus’”.

Besides feeling humiliated, students also frequently felt intimidated and threatened during their commute. One student described an experience going through a checkpoint:

“I once cried to the doctor because of my experience at the checkpoint with a soldier. He talked to me in a different language, Hebrew, that he knows I don’t understand, and we talked back and forth and then he said, ‘You are a stupid individual’. He was screaming and not talking in the microphone through the glass so I couldn’t hear, and even if I heard him, I wouldn’t understand what he’s saying.”

Intimidation was not limited to Israeli soldiers, but also felt by other personnel once inside Jerusalem. One student noted, “Drivers inside Jerusalem sometimes take advantage of us and make us pay a lot. They control us, tell us where to get off. Buses are slower so sometimes we have to take these private cars that we don’t know and they take advantage [of us]”.

### Arbitrariness

In all focus groups, the theme of arbitrariness arose spontaneously and remained a central theme. Students often expressed their frustration with the unpredictability of the permit application process and checkpoint crossing. Discussing the lack of transparency in the reasoning behind permit rejection, one student said, “The criteria are just not clear, […] it seems random, mood-dependent”. Another student expanded further, “The criteria are not clear—some people are active in Hamas and they get it [the permit]. None of us here in this [focus group] have active political activities and we didn’t get the permit but others are very active and they get permits normally. So the criteria just aren’t clear”. Students whose permits were rejected for unknown reasons sometimes reached out to advocacy organizations, such as Physicians for Human Rights Israel, which served as liaison between the student and Israeli authorities.

The reference to ‘mood’ was recurrent in descriptions of interactions with Israeli soldiers, especially at checkpoints. One student said, “They sometimes close it [checkpoint] randomly and then they want to sleep or eat or be on Facebook—and we just wait until they open again”. Due to this perception of arbitrariness in their daily lives, students expressed a pervasive sense of insecurity and uncertainty. Many students described how difficult it was not to be able to predict their commute times to work, since this was dependent on their experience at the checkpoints. Some students felt that this arbitrariness was a demonstration of power on the part of the occupying force: “They want us to remember that we’re always under occupation, and that they can do whatever they want with us”.

### Effect on medical education

Students emphasized that being trained at Al-Makassed Hospital was an imperative part of their medical education and those that did not get the opportunity to train there were at a learning disadvantage, especially in certain specialties, such as Pediatrics and Obstetrics and Gynecology. Students noted that Al-Makassed has more complex cases and the best teachers, since most of their medical school professors and clinical clerkship directors are based there. Comparing it to other hospitals in the West Bank, one student explained, “For Pediatrics, we felt a huge difference [in our training] in other hospitals compared to Al-Makassed, where they have formal bedside teaching. We only had one doctor in Hebron responsible for us, no lectures, no exams, no teaching. There wasn’t a system”. For students whose permits did not allow them to go to Jerusalem, they relied on self-study and learning from the experience of students who were able to train at Al-Makassed, but stressed that this felt inadequate. For others, they expressed a desire to take a year abroad in order to gain access to better training, an opportunity that some students were also denied due to rejection of permits to leave the country. It was common for teachers to express that students who did not train at Al-Makassed were behind in their skills compared to their peers who had trained at Al-Makassed.

Students in each focus group talked specifically about how the commute to Jerusalem had a negative impact on their studies and clinical performance. In discussing the effect of the commute, one student said, “You go through the mistreatment and mess of the morning, and we reach the hospital here already tired and then the doctor asks, ‘What did you study, what didn’t you…’. You start your day in a bad psychological state and then that stays with you the whole day. Would we be able to study well in this condition? Of course not”.

The students also emphasized how their ability to concentrate on their studies was impacted by violence from the region’s political instability. Describing the University environment, one student said, “There is tear gas by the University where we live--it’s fire: there are always clashes and stress and shooting and throwing rocks and sound bombs and rubber bullets. One is carrying a book, but our minds are constantly on the look out outside. It is not easy to study in such circumstances. It’s a place for constant battle”. In addition to fatigue and sleep deprivation, students consistently expressed that the commute left little time for seeing friends and family and was financially burdensome.

## Discussion

The findings from this study identify some of the hardships that Palestinian medical students face when undergoing their clinical training in the West Bank and Jerusalem. Living and studying in an occupied territory characterized by permit and checkpoint regulations significantly hindered the students’ ability to access high quality medical training.

Medical students who had their permits to enter Jerusalem rejected were unable to train at Al-Makassed Hospital and were often forced to rely on clinical training in the West Bank, which they felt was less adequate. Most students who had their permits rejected could not identify a reason for their rejection, even upon inquiry or consultation with human rights advocacy groups. Indeed, students often felt that decisions about who was able to get a permit or which checkpoints were closed were arbitrary, leading to a constant feeling of uncertainty in their daily lives. Almost all students surveyed, regardless of permit or ID status, expressed facing long delays at checkpoints and taking long routes around the Separation Wall to travel short distances. Students felt humiliated and intimidated by Israeli soldiers throughout the entire process of trying to get to Jerusalem and other hospitals.

Consistent with other literature on the challenges of receiving an education under occupation, focus groups highlighted how the emotional and psychological burden of the occupation specifically affected them as students, as it was hard to concentrate on their studies due to a stressful commute and surrounding political instability and violence [10]. Their quality of life was impacted with little time for sleep or social activity. In the face of these challenges, Palestinian medical students exhibited extraordinary resilience, resourcefulness, and dedication to their education.

Obstacles to accessing education in the occupied Palestinian territories are not limited to medical students. Since the beginning of the Israeli occupation, confrontation and tension surrounding Palestinian education has been pervasive at all educational levels [11]. Given the importance of education to the Palestinian identity, the obstruction and control of their education by Israeli authorities have been viewed as a means of exerting control and power over the Palestinian people [11]. Indeed, the location of Al-Quds University, which translates to “Jerusalem University”, was physically cut off from Jerusalem with the construction of the West Bank Separation Wall, which began in 2002. Al-Makassed Hospital not only carries significance for medical students because of its value as an exemplary teaching hospital, but it also serves as one of the last links between Al-Quds University and Jerusalem, which is both politically and symbolically significant to the Palestinian identity.

The results from this study emphasize the significant impact of political and military occupation on the daily lives and identities of this highly educated Palestinian population. These findings are consistent with a previous study that explored the challenges faced by Palestinian health workers living and working in the West Bank [6, 12]. There was significant overlap in the themes identified among medical students and health care professionals, specifically the emphasis placed on delays, searches and mistreatment. However, this is the first study to illustrate the hardships that Palestinian medical students specfically endure and how this affects their education. Indeed, compared to more seasoned physicians, medical students are more vulnerable to daily stresses of medical training and living under occupation, with unknown impacts on their training and career trajectory [13]. Further, even after completing their residency, the hardships of training will continue with little access to continued medical education in subspecialties and no system in place to ensure that physicians’ skillsets are retained [3].

The delays and obstacles that medical students and health workers experience reflect more broadly on the barriers that the general Palestinian population face in accessing health care. The construction of the West Bank Separation Wall has imposed an almost complete separation of the advanced hospitals in East Jerusalem from the population they are meant to serve [7]. In the town of Abu Dis, where Al-Quds University is located, the average time for an ambulance to travel to the nearest hospitals in Jerusalem increased from approximately 10 min to over one hour and 50 min after the Wall was constructed [14]. In 2005, 18 % of those seeking treatment at emergency departments in the West Bank were delayed by checkpoints or occupation-related detours [15]. Further contributing to poor health in the region, there is an extreme shortage of trained physicians in the OPT, with only 1.8 physicians for every 1000 people in the West Bank and 2.6 per 1000 in the Gaza Strip [16]. More concerning is the tendency of trained Palestinian physicians to emigrate, further exacerbating scarce access to trained medical professionals [17].

The findings from this study suggest that many medical students living and studying in the OPT receive sub-optimal training due to ambiguous permit rules, long delays at checkpoints and the psychological burden of the process. In a setting in which there is regular violence and many health indicators are already poor [6, 12], it is even more imperative to have well trained medical professionals [1]. International educational partnerships that leverage creative educational tools, such as the OxPal Medlink, an internet-based teaching program that connects clinical tutors at Oxford University with medical students in the West Bank, should be encouraged and continued in order to address gaps in students’ education [18]. However, given the political context of the OPT, larger institutional changes are needed to address the unjust hardships that medical professionals-in-training must undergo to receive a high-quality education. External pressure should be placed on occupying forces to uphold international laws and codes of ethics, as impeding movement of health workers violates the fourth Geneva Convention, which guarantees medical personnel respect and protection in occupied territories and military zones [19]. Indeed, a greater response is warranted to the repeated appeals from educational institutions in Palestine calling for financial support to address the dire conditions of facilities and the dearth of resources [10]. Policies are needed that encourage transparency in the permit process and ease of access for medical students crossing checkpoints to their sites of clinical training. Ultimately, a larger context of peace and sovereignty will be required to build an effective health care system in which Palestinian medical students can pursue adequate clinical training that will prepare them to provide healthcare for their vulnerable population.

### Limitations

Several limitations exist in this study. First, only 35 5th and 6th year students participated in the focus groups. One reason for low participation could be the busy schedules of the medical students. Additionally, given the content of the discussions, students could have been concerned for their safety should their identifies be discovered by authorities. No fourth year students participated in the focus groups, likely due to the fact that they had no classes at Al-Quds University during the time period of study recruitment and enrollment. Further, it is plausible that focus group discussions were biased towards those participants experiencing permit or commute difficulties. Students' answers could also have been influenced by the fact that the interviewer was not from Palestine. Participant recruitment was done through a convenience sample and it is plausible that there are some perspectives that have not been captured.

## Conclusions

Our findings suggest that medical students living and studying in the occupied Palestinian territories receive sub-optimal training due to ambiguous permit rules, barriers at checkpoints, and the psychological burden of the process. These results highlight the impact that military occupation has on the education and quality of life of Palestinian medical students in a setting in which there is regular violence and many health indicators are already poor. New transparent policies and a larger context of peace and sovereignty will be required to build an effective health care system in which Palestinian medical students can pursue adequate clinical training that will prepare them to care for their people in a resource-poor and war-torn setting.

## Additional file

Additional file 1:
**Focus Group Guiding Questions.** (DOCX 17 kb)
