# Supplementary material for: Attitudes of Palestinian medical students on the geopolitical barriers to accessing hospitals for clinical training: a qualitative study
Source: Confl Health. 2016 Feb 24;10:5. doi: 10.1186/s13031-016-0067-8 (PMC4765224; doi:10.1186/s13031-016-0067-8)
Supplement: Additional file 1: — Focus Group Guiding Questions. (DOCX 17 kb) [file 13031_2016_67_MOESM1_ESM.docx]

**Attitudes of Palestinian Medical Students on the Geopolitical Barriers to Accessing Hospitals for Clinical Training: A Qualitative Study**

**FOCUS GROUP GUIDING QUESTIONS**

**Demographic information:**

- Ages:
- Genders:
- Year of medical school:
- City currently living in:
- Hospitals trained at so far and their locations:
- Jerusalem ID or West Bank ID:

**Permits**

- Have you ever had to apply for access to a hospital site of training?
  - Please describe the process.
  - How long did it take?
  - Did you submit the application for a permit yourself or did someone do it for you?
  - How long did you have access to this hospital?
- Have any of you ever been denied access to a hospital site of training?
  - For what reason?
  - Did you try to appeal the decision? If so, how? What was the outcome?

**Your trip to the hospital**

- Which hospitals have been the most difficult for you to access so far?
- How long does your commute to that hospital take?
- How far is the distance from you?
- Do you have to cross checkpoints on your way to the hospitals?
  - How many?
  - Describe the process of crossing checkpoints
- Have you ever had to change your place of living, temporarily or permanently, to more easily access your hospital?
- Have you ever chosen not to rotate at a certain hospital because it was too difficult to access?

**IDs and Access**

- Describe what kind of ID card you carry and how this affects your access to hospitals.
- Given your ID status, is there a difference for you in trying to access a hospital in Jerusalem versus a hospital in the West Bank? Please describe if so.

**Effect on education and quality of life**

- Do you feel that your experiences accessing hospital sites of training have affected your medical education?
  - Did your experience accessing a hospital or returning home from a hospital affect your education at that hospital? Did it affect how early you got there or how late you returned?
- Do you feel that your experiences accessing hospital sites of training affect your quality of life? How so?

**Open-ended**

- Any additional thoughts?
